# The Role of Primary Tumor Resection in De Novo Bone‐Only Metastatic Breast Cancer: A Propensity Score‐Matched Analysis

**DOI:** 10.1002/jso.70277

**Published:** 2026-04-29

**Authors:** Bin Wang, Shasha Liu, Zhidong Huang, Jinhui Wang, Xirui Zhou, Shuo Li, Jianan Chen, Hong Liu

**Affiliations:** ^1^ The Second Surgical Department of Breast Cancer, Cancer Institute and Hospital, National Clinical Research Center for Cancer, Key Laboratory of Breast Cancer Prevention and Therapy, Tianjin's Clinical Research Center for Cancer Tianjin Medical University Tianjin China; ^2^ Department of Clinical Sciences H. Lee Moffitt Cancer Center & Research Institute Tampa Florida USA

**Keywords:** metastatic breast cancer, primary tumor resection, propensity score matching, SEER program, survival analysis

## Abstract

**Objective:**

To evaluate the impact of primary tumor resection (PTR) on overall survival (OS) and cancer‐specific survival (CSS) in women with de novo bone‐only metastatic breast cancer (MBC).

**Methods:**

Women diagnosed with de novo bone‐only metastatic breast cancer between 2010 and 2017 were identified from the Surveillance, Epidemiology, and End Results (SEER) database. Patients were included if they had bone metastasis at diagnosis but no lung, liver, or brain involvement. Primary tumor resection was defined as any cancer‐directed breast surgery. Propensity scores were calculated using multivariable logistic regression incorporating demographics, tumor features, and treatment factors, followed by 1:1 nearest‐neighbor matching (caliper = 0.2 SD). Kaplan–Meier, Cox proportional hazards, and restricted mean survival time (RMST) analyses assessed overall survival (OS) and cancer‐specific survival (CSS). Time‐dependent ROC curves (timeROC) evaluated model discrimination. Determinants of PTR were examined using multivariable logistic regression.

**Results:**

A total of 3296 women with de novo bone‐only metastatic breast cancer were included (1252 with PTR; 2044 without). After 1:1 matching, 2002 well‐balanced patients remained (1001 per group). Median OS and CSS were significantly longer among patients undergoing surgery (OS: 69 vs. 39 months; CSS: 76 vs. 41 months; both *p* < 0.0001). PTR remained an independent predictor of improved OS (HR = 0.54, 95% CI 0.48–0.60, *p* < 0.001), with consistent benefit across subgroups. RMST differences (surgery − no surgery) increased with time, reaching 9.05 months for OS and 8.79 months for CSS at 60 months. Model discrimination was acceptable (AUCs for OS: 0.753, 0.734, 0.717 at 1–, 3–, and 5‐years). Radiation therapy was positively associated with PTR (OR = 1.37, 95% CI 1.12–1.68, *p* = 0.002), whereas well/moderate grade predicted lower odds (OR = 0.80, 95% CI 0.66–0.99, *p* = 0.036).

**Conclusions:**

In this large SEER‐based propensity‐matched analysis, primary tumor resection was associated with significantly improved overall and cancer‐specific survival among women with de novo bone‐only metastatic breast cancer. These findings suggest that selected patients with isolated bone metastasis may benefit from locoregional surgery, warranting further prospective validation.

**Abbreviations**
AUC: area under the curve
BC: breast cancer
BOBM: bone‐only breast metastasis
CI: confidence interval
CSS: cancer‐specific survival
ER: estrogen receptor
HER2: human epidermal growth factor receptor 2
HR: hazard ratio
LRT: locoregional treatment
MBC: metastatic breast cancer
*N* stage: nodal stage
OR: odds ratio
OS: overall survival
PR: progesterone receptor
PS: propensity score
PSM: propensity score matching
PTR: primary tumor resection
*R*
: R programming language
RMST: restricted mean survival time
ROC: receiver operating characteristic
SEER: surveillance, epidemiology, and end results
STROBE: strengthening the reporting of observational studies in epidemiology.
TNBC: triple‐negative breast cancer
*T* stage: tumor stage
USD: United States dollars

## 1 Introduction

Breast cancer remains one of the most prevalent malignancies and a major cause of cancer‐related mortality among women worldwide [1]. Although de novo metastatic breast cancer (MBC) has traditionally been considered incurable, advances in systemic therapy have significantly extended survival, leading to renewed discussion about the role of local treatment in this setting [2, 3]. The management of MBC remains primarily systemic, while surgery of the primary tumor has historically been limited to palliation of symptoms such as ulceration or bleeding [4].

Emerging observational studies have suggested that primary tumor resection (PTR) may offer a survival advantage in selected patients with metastatic disease, possibly through reducing tumor burden, limiting further dissemination, or enhancing systemic therapy efficacy [5]. However, results from randomized controlled trials (RCTs) have been inconsistent, and the benefit of PTR remains controversial [6]. This uncertainty is particularly relevant for patients with bone‐only metastases, a subgroup characterized by a more indolent disease course, lower tumor burden, and longer expected survival compared to those with visceral metastases [7, 8].

To clarify the potential role of PTR in this distinct population, we conducted a large population‐based study using data from the Surveillance, Epidemiology, and End Results (SEER) program. By applying propensity score matching (PSM) to minimize treatment‐selection bias, we aimed to evaluate the association between PTR and overall survival (OS) as well as cancer‐specific survival (CSS) in women with de novo bone‐only MBC. This study seeks to provide contemporary, real‐world evidence to inform individualized surgical decision‐making for these patients.

## 2 Methods

### 2.1 Data Source

We used data from the SEER program of the U.S. National Cancer Institute. Specifically, we accessed the SEER Research Plus, 17 Registries, Nov 2022 submission (diagnosis years 2000–2020) via SEER*Stat v9.0.4. SEER provides de‐identified, population‐based cancer incidence and vital status information covering ~30% of the U.S. population; therefore, institutional review board approval and informed consent were not required.

### 2.2 Study Population

We identified female patients diagnosed with de novo MBC between 2010 and 2017 using the SEER database. Eligible cases met the following criteria: a malignant primary breast tumor (ICD‐O‐3 topography C50.x), bone metastasis only at diagnosis, one reportable primary tumor, known survival time, and complete follow‐up for vital status. Patients with liver, lung, brain, or other distant metastases, those identified only through autopsy or death certificate, or with missing key variables were excluded. To reduce potential treatment‐selection bias, we further restricted the cohort to patients who received chemotherapy and did not undergo surgery for bone metastasis. Histologic subtype was limited to adenocarcinoma to ensure diagnostic homogeneity. The primary exposure was PTR, defined using the SEER variable “Surgery of Primary Site” (any cancer‐directed breast surgery vs none). Patients were divided into PTR and no‐PTR groups for subsequent comparative and propensity‐score‐matched analyses.

### 2.3 Variables and Outcomes

Baseline covariates were prespecified according to clinical relevance and data availability and mirror the variables used in the analysis code: age at diagnosis (young breast cancer, < 40 years vs ≥ 40 years), race (American Indian/Alaska Native, Asian or Pacific Islander, Black, White, Unknown), marital status (married vs unmarried), county‐level income (≤$50 000; $50 001–$74 999; ≥$75 000), residence (rural/urban), histologic grade (poor/undifferentiated; well/moderate; unknown), *T* stage, *N* stage, laterality (left/right/unknown), radiation (any vs none), hormone‐receptor subtype (triple‐negative breast cancer [TNBC] vs non‐TNBC, defined by ER/PR/HER2 in SEER), and year of diagnosis (2010–2017). In descriptive and propensity models, “Unknown” was retained as an explicit category; no imputation was performed. Primary outcomes were OS—time from diagnosis to death from any cause—and CSS—time from diagnosis to death attributed to breast cancer per SEER cause‐specific death classification. Patients alive at the last follow‐up were censored.

### 2.4 Propensity Score Methodology

To mitigate baseline imbalances, we estimated each patient's propensity score (PS) for receiving PTR using multivariable logistic regression including all prespecified covariates: year of diagnosis, race, grade, laterality, *T* stage, *N* stage, radiation, hormone‐receptor subtype (TNBC vs non‐TNBC), age group, marital status, income, and residence (rural/urban). We then performed 1:1 nearest‐neighbor matching without replacement on the logit of the PS with a caliper of 0.2 SD, targeting the average treatment effect on the treated (ATT), implemented with the MatchIt package. Covariate balance was assessed using standardized mean differences (SMDs) with SMD ≤ 0.10 considered acceptable, tabulated pre‐/post‐matching, and visualized using kernel‐density PS distributions. The matched dataset was exported for downstream analyses.

### 2.5 Survival Analyses

In both the unmatched and matched cohorts, we generated Kaplan–Meier curves for OS and CSS and compared groups using two‐sided log‐rank tests. We fitted Cox proportional hazards models to estimate hazard ratios (HRs) with 95% confidence intervals (CIs) for the association between PTR and survival. For OS, we conducted (i) univariable models for each covariate and (ii) a multivariable model adjusting for all prespecified covariates. The proportional hazards assumption was examined using Schoenfeld residuals and log–log plots. Predefined subgroup analyses were carried out in the matched cohort across strata of age, race, marital status, income, residence, grade, *T* stage, *N* stage, laterality, radiation, hormone‐receptor subtype, and year of diagnosis; subgroup‐specific HRs (surgery vs no surgery) were displayed in a forest plot, and interaction with PTR was tested via likelihood‐ratio tests comparing models with and without interaction terms.

### 2.6 RMST, Discrimination, and Treatment Selection

Beyond the primary survival analyses, we further quantified the average treatment benefit by estimating restricted mean survival time (RMST) differences between the surgery and no‐surgery groups at 12, 24, 36, 48, and 60 months using survRM2, and evaluated model discrimination using time‐dependent ROC curves (timeROC) derived from the multivariable Cox model, reporting AUCs at 1, 3, and 5 years for both OS and CSS; and investigated factors associated with the receipt of PTR through a multivariable logistic regression in the matched cohort, incorporating the same covariates as the PS model. Levels labeled as “Unknown” were treated as missing, and a complete‐case approach was applied. Results were expressed as adjusted odds ratios (ORs) with 95% CIs and likelihood‐ratio global *p*‐values for multi‐level factors.

### 2.7 Statistical Software

All analyses were conducted in R 4.5.0. Packages included MatchIt, cobalt, survival, survminer, broom, dplyr, tidyr, forcats, ggplot2, gt, survRM2, and timeROC. Two‐sided *α* = 0.05 defined statistical significance.

## 3 Results

### 3.1 Patient Selection and Cohort Construction

**Figure 1:**
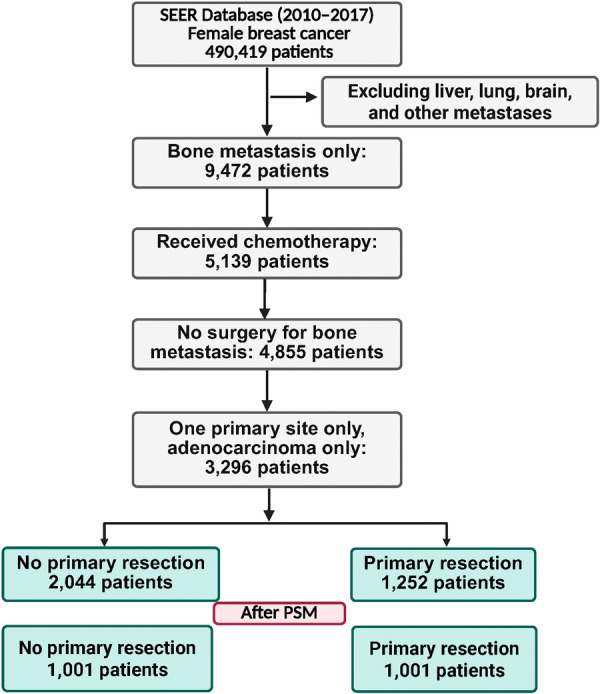
Study cohort selection flowchart. Flow diagram illustrating patient selection from the SEER database (2010–2017). Among 490 419 female breast cancer patients, those with liver, lung, brain, or other distant metastases were excluded, leaving 9472 patients with bone metastasis only. After restricting to patients who received chemotherapy (*n* = 5,139) and excluding those who underwent surgery for bone metastasis, 4855 patients remained. Limiting to cases with one primary site and histologically confirmed adenocarcinoma yielded 3,296 eligible patients. Of these, 2044 did not undergo primary tumor resection (PTR), and 1252 did. After 1:1 propensity score matching (nearest neighbor, caliper as described in Methods), 1001 matched pairs were included for subsequent analyses.

**Figure 2:**
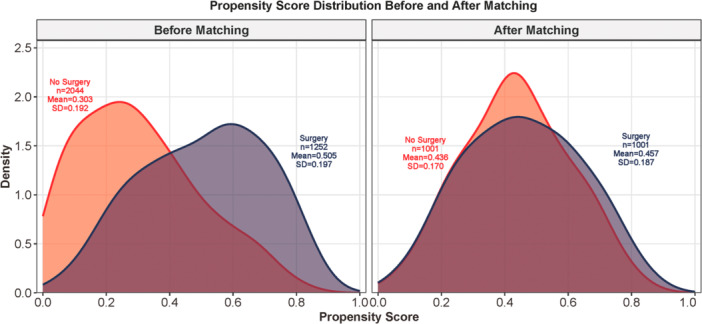
Propensity score distribution before and after matching. Kernel density plots of the estimated propensity scores for the surgery and no‐surgery groups before (left) and after (right) 1:1 propensity score matching (nearest neighbor, caliper as in Methods). Before matching, distributions were more separated (No surgery: *n* = 2,044; mean = 0.303, SD = 0.192; Surgery: *n* = 1,252; mean = 0.505, SD = 0.197). After matching, overlap improved (No surgery: *n* = 1,001; mean = 0.436, SD 0.170; Surgery: *n* = 1,001; mean = 0.457, SD = 0.187).

The study cohort was assembled according to the flowchart shown in Figure 1. From 490 419 female breast cancer patients recorded in SEER between 2010 and 2017, we identified 9472 patients with bone metastasis only after excluding those with liver, lung, brain, or other distant metastases. Of these, 5139 patients received chemotherapy, and 4855 had no record of surgery for bone lesions. After restricting to cases with a single primary site and adenocarcinoma histology, 3296 patients were eligible for analysis. Among them, 1252 (38.0%) underwent PTR, did not. Propensity scores differed markedly between groups before matching (mean ± SD: 0.505 ± 0.197 for surgery vs. 0.303 ± 0.192 for no surgery). After 1:1 nearest‐neighbor PSM, 2002 patients remained (1001 per group), showing substantial overlap in PS distributions (surgery: 0.457 ± 0.187; no surgery: 0.436 ± 0.170) (Figure 2).

### 3.2 Baseline Characteristics Before and After Matching

Before matching (Table 1), groups differed in several covariates, notably *N* stage, *T* stage, marital status, and receipt of radiation (all *p* < 0.05), with large SMDs for radiation (SMD 0.57) and unknown *T*, *N* categories. After matching (Table 2), baseline characteristics were comparable across groups, with small SMDs (generally ≤ 0.07) and non‐significant level tests for most variables; the kernel‐density plots further confirmed improved overlap (Figure 2).

**Table 1:** Baseline characteristics before propensity score matching.

Variable	Surgery, *n* (%)	No surgery, *n* (%)	*p* value	SMD
Age group				
< 40 years	210 (16.8%)	208 (10.2%)		0.19
≥ 40 years	1042 (83.2%)	1836 (89.8%)	< 0.001	0.19
Grade				
Poor/Undifferentiated	549 (43.8%)	567 (27.7%)	< 0.001	0.34
Well/Moderate	578 (46.2%)	888 (43.4%)		0.05
Unknown	125 (10.0%)	589 (28.8%)		0.49
Hormone receptor				
non‐TNBC	1086 (86.7%)	1755 (85.9%)	0.51	0.03
TNBC	166 (13.3%)	289 (14.1%)		0.03
Income (USD)				
≤ 50 000	86 (6.9%)	138 (6.8%)	0.063	0
50 001–74 999	507 (40.5%)	747 (36.5%)		0.08
≥ 75 000	659 (52.6%)	1159 (56.7%)		0.08
Laterality				
Left	664 (53.0%)	981 (48.0%)	< 0.001	0.1
Right	583 (46.6%)	946 (46.3%)		0.01
Unknown	5 (0.4%)	117 (5.7%)		0.31
Marital status				
Married	664 (53.0%)	989 (48.4%)	0.011	0.09
Unmarried	588 (47.0%)	1055 (51.6%)		0.09
*N* stage				
N0	172 (13.7%)	448 (21.9%)	< 0.001	0.21
*N* positive	1049 (83.8%)	1390 (68.0%)		0.38
Unknown	31 (2.5%)	206 (10.1%)		0.32
Race				
American Indian/Alaska Native	10 (0.8%)	11 (0.5%)	0.391	0.03
Asian or Pacific Islander	106 (8.5%)	152 (7.4%)		0.04
Black	176 (14.1%)	320 (15.7%)		0.04
White	958 (76.5%)	1554 (76.0%)		0.01
Unknown	2 (0.2%)	7 (0.3%)		0.04
Radiation				
No	529 (42.3%)	1422 (69.6%)	< 0.001	0.57
Yes	723 (57.7%)	622 (30.4%)		0.57
Rural/Urban				
Rural	165 (13.2%)	231 (11.3%)	0.12	0.06
Urban	1087 (86.8%)	1813 (88.7%)		0.06
*T* stage				
T1/T2	658 (52.6%)	777 (38.0%)	< 0.001	0.3
T3/T4	543 (43.4%)	908 (44.4%)		0.02
Unknown	51 (4.1%)	359 (17.6%)		0.44
Year of diagnosis				
2010	177 (14.1%)	170 (8.3%)	< 0.001	0.19
2011	176 (14.1%)	213 (10.4%)		0.11
2012	174 (13.9%)	199 (9.7%)		0.13
2013	136 (10.9%)	254 (12.4%)		0.05
2014	130 (10.4%)	217 (10.6%)		0.01
2015	160 (12.8%)	341 (16.7%)		0.11
2016	147 (11.7%)	307 (15.0%)		0.1
2017	152 (12.1%)	343 (16.8%)		0.13

**Table 2:** Baseline characteristics after propensity score matching.

Variable	Surgery, *n* (%)	No surgery, *n* (%)	*p* value	SMD
Age group				
< 40 years	145 (14.5%)	130 (13.0%)		0.04
≥ 40 years	856 (85.5%)	871 (87.0%)	0.363	0.04
Grade				
Poor/Undifferentiated	410 (41.0%)	382 (38.2%)	0.433	0.06
Well/Moderate	469 (46.9%)	494 (49.4%)		0.05
Unknown	122 (12.2%)	125 (12.5%)		0.01
Hormone receptor				
non‐TNBC	869 (86.8%)	863 (86.2%)	0.744	0.02
TNBC	132 (13.2%)	138 (13.8%)		0.02
Income (USD)				
≤ 50 000	73 (7.3%)	67 (6.7%)	0.597	0.02
50 001–74 999	408 (40.8%)	392 (39.2%)		0.03
≥ 75 000	520 (51.9%)	542 (54.1%)		0.04
Laterality				
Left	529 (52.8%)	532 (53.1%)	0.418	0.01
Right	467 (46.7%)	459 (45.9%)		0.02
Unknown	5 (0.5%)	10 (1.0%)		0.06
Marital status				
Married	511 (51.0%)	515 (51.4%)	0.893	0.01
Unmarried	490 (49.0%)	486 (48.6%)		0.01
*N* stage				
*N*0	162 (16.2%)	157 (15.7%)	0.954	0.01
*N* positive	809 (80.8%)	814 (81.3%)		0.01
Unknown	30 (3.0%)	30 (3.0%)		0
Race				
American Indian/Alaska Native	7 (0.7%)	9 (0.9%)	0.796	0.02
Asian or Pacific Islander	90 (9.0%)	76 (7.6%)		0.05
Black	145 (14.5%)	142 (14.2%)		0.01
White	757 (75.6%)	772 (77.1%)		0.04
Unknown	2 (0.2%)	2 (0.2%)		0
Radiation				
No	505 (50.4%)	542 (54.1%)	0.107	0.07
Yes	496 (49.6%)	459 (45.9%)		0.07
Rural/Urban				
Rural	126 (12.6%)	121 (12.1%)	0.786	0.02
Urban	875 (87.4%)	880 (87.9%)		0.02
*T* stage				
T1/T2	488 (48.8%)	479 (47.9%)	0.761	0.02
T3/T4	463 (46.3%)	465 (46.5%)		0
Unknown	50 (5.0%)	57 (5.7%)		0.03
Year of diagnosis				
2010	134 (13.4%)	120 (12.0%)	0.944	0.04
2011	125 (12.5%)	121 (12.1%)		0.01
2012	119 (11.9%)	125 (12.5%)		0.02
2013	119 (11.9%)	121 (12.1%)		0.01
2014	115 (11.5%)	104 (10.4%)		0.04
2015	135 (13.5%)	142 (14.2%)		0.02
2016	119 (11.9%)	121 (12.1%)		0.01
2017	135 (13.5%)	147 (14.7%)		0.03

### 3.3 Survival Outcomes Before and After PSM

**Figure 3:**
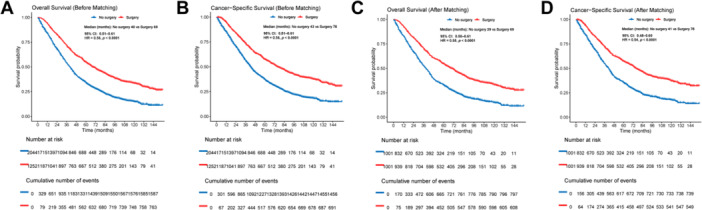
Overall and cancer‐specific survival curves before and after matching. Kaplan–Meier survival curves comparing surgery versus no surgery. Hazard ratios (HRs) are from Cox models, and *p‐*values from log‐rank tests. (A) OS before matching: median survival 40 months (no surgery) versus 69 months (surgery); HR = 0.56, *p* < 0.0001. (B) CSS before matching: median 43 versus 76 months; HR = 0.56, *p* < 0.0001. (C) OS after matching: median 39 versus 69 months; HR = 0.55, *p* < 0.0001. (D) CSS after matching: median 41 versus 76 months; HR = 0.54, *p* < 0.0001. Numbers at risk and cumulative events are shown below each panel.

Kaplan–Meier analyses showed consistently superior survival among patients who underwent surgery both before and after matching (Figure 3). Before matching, median OS was 69 months with surgery versus 40 months without; median CSS was 76 versus 43 months (both log‐rank *p* < 0.0001; HRs = 0.55–0.56). After matching, the survival advantage persisted: median OS 69 versus 39 months and median CSS 76 versus 41 months (both *p* < 0.0001; HRs = 0.54–0.55). In the matched cohort (Table 4), 1−, 3−, 5‐year OS rates were 92.5%, 70.3%, 54.5% with surgery versus 82.9%, 52.4%, 32.8% without; corresponding CSS rates were 93.6%, 72.2%, 57.3% versus 84.2%, 54.7%, 35.5%.

### 3.4 Cox Regression Analyses

**Figure 4:**
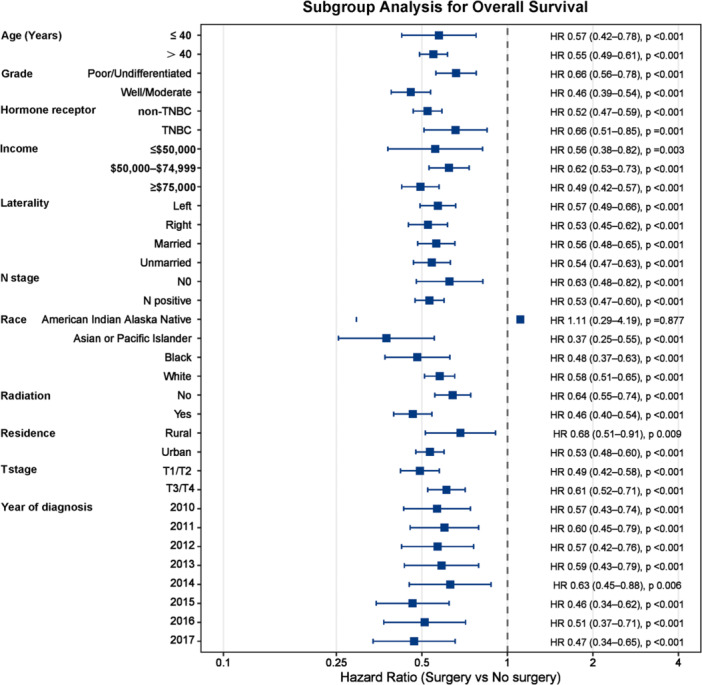
Subgroup analysis for overall survival in the matched cohort. Forest plot of subgroup Cox analyses showing HRs for surgery versus no surgery (vertical dashed line at HR = 1). Subgroups include age, grade, hormone receptor, income, laterality, marital status, *N* stage, race, radiation, residence, *T* stage, and year of diagnosis. Most subgroups favored surgery (HR < 1) with non‐overlapping 95% Cis.

Univariable and multivariable Cox models (Table 3) identified TNBC (HR = 2.75, 95% CI 2.37–3.19, *p* < 0.001), T3–T4 stage (HR = 1.23, 1.10–1.37, *p* < 0.001), and *N*‐positive disease (HR = 1.18, 1.01–1.37, *p* = 0.034) as independent predictors of worse OS, whereas well, moderate grade was protective (HR = 0.85, 0.75–0.95, *p* = 0.004). Age < 40 years was not independently associated with OS after adjustment (HR = 0.87, 0.74–1.02, *p* = 0.094). Calendar year showed an improving prognosis compared with 2010 (e.g., 2014 HR = 0.63, 0.51–0.79, *p* < 0.001). Subgroup analyses in the matched cohort demonstrated a broadly consistent OS benefit of surgery across prespecified strata (Figure 4), with most subgroup HRs favoring surgery and 95% CIs not crossing unity.

**Table 3:** Univariable and multivariable Cox regression analysis for overall survival after matching.

Variable	Category	Univariable HR (95% CI)	*p*‐value	Multivariable HR (95% CI)	*p*‐value
Age (Years)	≥ 40 years	1.00 (ref)		1.00 (ref)	
	< 40 years	0.80 (0.68–0.93)	0.005	0.87 (0.74–1.02)	0.094
Grade	Poor/Undifferentiated	1.00 (ref)		1.00 (ref)	
	Well/Moderate	0.76 (0.68–0.85)	< 0.001	0.85 (0.75–0.95)	0.004
	Unknown	0.95 (0.80–1.12)	0.53	0.85 (0.72–1.01)	0.065
Hormone receptor	non‐TNBC	1.00 (ref)		1.00 (ref)	
	TNBC	2.80 (2.44–3.23)	< 0.001	2.75 (2.37–3.19)	< 0.001
Income (USD)	≤ 50 000	1.00 (ref)		1.00 (ref)	
	50 001–74 999	0.92 (0.75–1.13)	0.444	1.08 (0.85–1.37)	0.549
	≥ 75 000	0.80 (0.65–0.98)	0.03	0.95 (0.74–1.22)	0.688
Laterality	Left	1.00 (ref)			
	Right	0.93 (0.84–1.03)	0.166		
	Unknown	1.59 (0.94–2.70)	0.086		
Marital status	Married	1.00 (ref)			
	Unmarried	1.11 (1.00–1.23)	0.056		
*N* stage	*N*0	1.00 (ref)		1.00 (ref)	
	*N* positive	1.17 (1.01–1.35)	0.035	1.18 (1.01–1.37)	0.034
	Unknown	1.27 (0.92–1.75)	0.151	1.02 (0.72–1.45)	0.915
Race	American Indian/Alaska Native	1.00 (ref)			
	Asian or Pacific Islander	1.03 (0.52–2.04)	0.921		
	Black	1.69 (0.87–3.29)	0.122		
	White	1.15 (0.60–2.22)	0.674		
	Unknown	0.96 (0.21–4.45)	0.96		
Radiation	No	1.00 (ref)			
	Yes	0.96 (0.87–1.07)	0.458		
Rural/Urban	Rural	1.00 (ref)		1.00 (ref)	
	Urban	0.78 (0.67–0.91)	0.002	0.83 (0.69–1.00)	0.056
*T* stage	T1/T2	1.00 (ref)		1.00 (ref)	
	T3/T4	1.32 (1.18–1.47)	< 0.001	1.23 (1.10–1.37)	< 0.001
	Unknown	1.13 (0.89–1.43)	0.323	0.93 (0.72–1.20)	0.555
Year of diagnosis	2010	1.00 (ref)		1.00 (ref)	
	2011	0.96 (0.79–1.16)	0.646	0.94 (0.78–1.14)	0.546
	2012	0.87 (0.72–1.07)	0.182	0.89 (0.73–1.09)	0.27
	2013	0.85 (0.69–1.04)	0.109	0.83 (0.68–1.02)	0.081
	2014	0.66 (0.53–0.82)	< 0.001	0.63 (0.51–0.79)	< 0.001
	2015	0.77 (0.63–0.94)	0.01	0.78 (0.63–0.95)	0.014
	2016	0.79 (0.64–0.97)	0.026	0.79 (0.64–0.98)	0.033
	2017	0.72 (0.58–0.89)	0.002	0.74 (0.60–0.92)	0.006
Primary tumor resection	No surgery	1.00 (ref)		1.00 (ref)	
	Surgery	0.55 (0.50–0.61)	< 0.001	0.54 (0.48–0.60)	< 0.001

**Table 4:** 1‐, 3‐, and 5‐year OS and CSS rates in the matched cohort.

Endpoint	Group	1‐year survival (%)	3‐year survival (%)	5‐year survival (%)
CSS	No surgery	84.2% (82.0%–86.5%)	54.7% (51.7%–58.0%)	35.5% (32.6%–38.7%)
	Surgery	93.6% (92.0%–95.1%)	72.2% (69.4%–75.0%)	57.3% (54.3%–60.5%)
OS	No surgery	82.9% (80.6%–85.3%)	52.4% (49.4%–55.6%)	32.8% (30.0%–35.8%)
	Surgery	92.5% (90.9%–94.2%)	70.3% (67.5%–73.2%)	54.5% (51.5%–57.7%)

**Figure 5:**
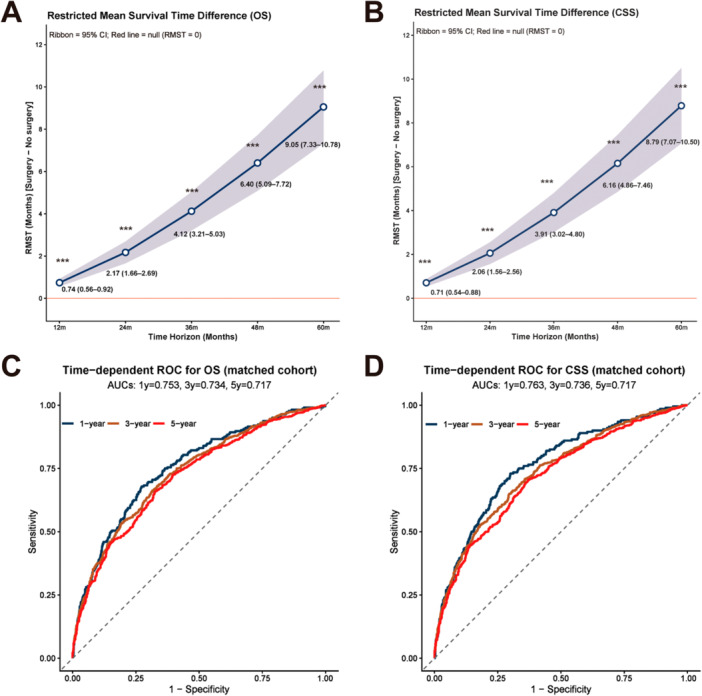
Restricted mean survival time (RMST) differences and time‐dependent ROC in the matched cohort. (A) OS—RMST difference (Surgery − No surgery): RMST benefit increased with time, from 0.74 months (95% CI 0.56–0.92) at 12 months to 9.05 months (7.33–10.78) at 60 months. (B) CSS—RMST difference: from 0.71 months (0.54–0.88) at 12 months to 8.79 months (7.07–10.50) at 60 months. Shaded ribbons indicate 95% CIs; the red horizontal line denotes the null (RMST = 0). (C) Time‐dependent ROC for OS: AUCs = 0.753 (1‐year), 0.734 (3‐year), 0.717 (5‐year). (D) Time‐dependent ROC for CSS: AUCs = 0.763 (1‐year), 0.736 (3‐year), 0.717 (5‐year).

Time‐restricted effectiveness was further supported by RMST differences (surgery − no surgery), which increased with follow‐up: OS 0.74 months at 12 months to 9.05 months at 60 months, and CSS 0.71 to 8.79 months (Figure 5A,B). Time‐dependent ROC curves showed acceptable discrimination of the survival models in the matched cohort: OS AUCs 0.753, 0.734, 0.717, and CSS AUCs 0.763, 0.736, 0.717 at 1, 3, 5 years (Figure 5C,D).

### 3.5 Factors Associated With Receiving Primary Tumor Resection

In multivariable logistic regression (Table 5), receipt of radiation was associated with higher odds of undergoing surgery (OR 1.37, 95% CI 1.12–1.68, *p* = 0.002), whereas well, moderate grade showed lower odds compared with poor, undifferentiated tumors (OR 0.80, 0.66–0.99, *p* = 0.036). Other sociodemographic and clinicopathologic variables—including age group, race, marital status, income, residence, laterality, and *T*, *N* stage—were not independently associated with surgery uptake (all global *p* values non‐significant).

**Table 5:** Multivariable logistic regression analysis for predictors of primary tumor resection.

Variable	Category	OR (95% CI)	*p*‐value	Global *p*
Age (Years)	≥ 40 years	1.00 (ref)		0.465
	< 40 years	1.11 (0.84–1.48)	0.465	
Race	American Indian/Alaska Native	1.00 (ref)		0.749
	Asian or Pacific Islander	1.59 (0.43–6.49)	0.49	
	Black	1.35 (0.37–5.46)	0.651	
	White	1.33 (0.38–5.27)	0.66	
Marital status	Married	1.00 (ref)		0.764
	Unmarried	0.97 (0.79–1.19)	0.764	
Income (USD)	≤ 50 000	1.00 (ref)		0.812
	50 001–74 999	0.86 (0.54–1.38)	0.535	
	≥ 75 000	0.85 (0.52–1.39)	0.527	
Residence	Rural	1.00 (ref)		0.924
	Urban	0.98 (0.68–1.42)	0.924	
Grade	Poor/Undifferentiated	1.00 (ref)		0.036
	Well/Moderate	0.80 (0.66–0.99)	0.036	
Hormone receptor	non‐TNBC	1.00 (ref)		0.74
	TNBC	0.95 (0.70–1.29)	0.74	
*T* stage	T1/T2	1.00 (ref)		0.181
	T3/T4	0.87 (0.71–1.07)	0.181	
*N* stage	N0	1.00 (ref)		0.39
	*N* positive	1.13 (0.86–1.48)	0.39	
Laterality	Left	1.00 (ref)		0.755
	Right	1.03 (0.85–1.26)	0.755	
Radiation	No	1.00 (ref)		0.002
	Yes	1.37 (1.12–1.68)	0.002	
Year of diagnosis	2010	1.00 (ref)		0.773
	2011	0.91 (0.60–1.37)	0.641	
	2012	0.94 (0.62–1.41)	0.754	
	2013	0.82 (0.54–1.22)	0.323	
	2014	1.09 (0.72–1.64)	0.695	
	2015	0.86 (0.59–1.26)	0.436	
	2016	0.81 (0.55–1.20)	0.296	
	2017	0.80 (0.54–1.18)	0.256	

## 4 Discussion

In this large, population‐based analysis of women with de novo bone‐only MBC, we found that PTR was independently associated with significantly improved overall and CSS. The survival advantage remained consistent after PSM and across clinically relevant subgroups. Our findings suggest that, in selected patients with isolated bone metastasis, surgical removal of the primary breast tumor may contribute to prolonged survival beyond the benefit of systemic therapy alone.

The role of locoregional surgery in de novo stage IV breast cancer remains a subject of ongoing debate [9, 10]. While systemic therapy continues to represent the cornerstone of management, emerging evidence suggests that resection of the primary tumor may offer potential survival advantages in selected subgroups [11, 12]. Multiple retrospective studies have demonstrated an association between PTR and improved outcomes, including a recent large‐scale analysis by Cui et al. showing significantly better overall and CSS among patients receiving surgery, particularly in those with bone‐only metastases [13]. Conversely, RCTs have yielded conflicting findings. The MF07‐01 trial [14] reported a 34% reduction in mortality with locoregional treatment and observed the greatest benefit in younger, hormone receptor–positive patients and those with solitary bone metastasis. In contrast, the Indian trial by Badwe et al. and the Austrian POSYTIVE study failed to demonstrate a consistent OS benefit, underscoring the complexity of patient selection and timing of surgery in this setting [6, 15].

These discrepancies likely reflect heterogeneity in metastatic patterns, biological subtypes, and systemic therapy regimens, as well as differences in trial design and inclusion criteria [7, 16]. As highlighted by Franceschini et al., surgery for the primary tumor should not be considered a universal intervention but rather a personalized strategy within a multidisciplinary framework, reserved for patients with limited metastatic burden, good performance status, and well‐controlled systemic disease [17]. From a biological standpoint, resection of the intact primary lesion may decrease circulating tumor cell release, reduce tumor‐induced immunosuppression, and enhance responsiveness to systemic therapy [10, 18]. Conversely, surgical stress and interruption of systemic treatment could theoretically accelerate metastatic progression, though this was not evident in our findings.

Our study contributes to this discussion by focusing exclusively on de novo bone‐only MBC, a biologically indolent subset often associated with prolonged survival and preserved functional status [6, 19]. By applying PSM to minimize treatment‐selection bias, we provide real‐world evidence that PTR may offer both disease control and survival benefit in this distinct group. These results reinforce the importance of individualized decision‐making and support the integration of locoregional surgery into multidisciplinary management for appropriately selected patients.

Several limitations of this study should be acknowledged. First, the SEER database lacks important clinical variables that may influence both treatment selection and survival outcomes, including the extent of bone metastases, performance status, comorbidities, and the timing of surgery relative to systemic treatment. These unmeasured factors may lead to the preferential selection of patients with more favorable disease characteristics for surgical intervention, thereby introducing potential selection bias. Although PSM was used to balance observed covariates, residual confounding cannot be excluded. Second, as a retrospective observational study, our findings are associative in nature and should not be interpreted as evidence of causality or as a replacement for RCTs [14, 20]. Rather, these results should be considered complementary to existing randomized evidence and primarily hypothesis‐generating. Third, the SEER database does not provide detailed information on systemic therapy, including endocrine therapy, HER2‐targeted therapy, and CDK4/6 inhibitors. Given that patients with bone‐only MBC are frequently hormone receptor–positive and treated with endocrine‐based regimens, the absence of these data limits our ability to fully account for treatment heterogeneity. Furthermore, the variable “receipt of chemotherapy” does not capture the complexity, sequencing, or effectiveness of modern systemic therapy. Advances in systemic treatments over the past decade may substantially influence survival outcomes and could partially explain the observed associations [21]. Therefore, the interaction between systemic therapy and surgical intervention cannot be adequately assessed in this study.

## 5 Conclusions

In conclusion, our study demonstrates that PTR is associated with improved survival in women with de novo bone‐only MBC. These findings suggest that local control of the primary tumor may have prognostic value in this biologically favorable subgroup and highlight the need for prospective, randomized studies to validate the survival benefit and define optimal patient selection criteria.

## Author Contributions

Bin Wang and Shasha Liu contributed equally to this work. Hong Liu conceived and supervised the study. Bin Wang, Shasha Liu, Shuo Li, and Zhidong Huang collected and curated the data. Jinhui Wang and Xirui Zhou performed statistical analyses and data visualization. Jianan Chen contributed to study design optimization, data interpretation, and manuscript revision. Bin Wang, Shasha Liu and Shuo Li drafted the initial manuscript. All authors reviewed and approved the final version of the manuscript.

## Funding

The authors have nothing to report.

## Ethics Statement

This study used de‐identified, publicly available data from the SEER database. Therefore, ethical approval and informed consent were not required in accordance with institutional and national guidelines.

## Consent

This study involved retrospective analysis of anonymized data from a public database; therefore, individual consent was not required.

## Conflicts of Interest

The authors declare that the research was conducted in the absence of any commercial or financial relationships that could be construed as a potential conflict of interest.

## 6 SYNOPSIS

In women with de novo bone‐only metastatic breast cancer, primary tumor resection was associated with survival benefit, supporting a potential role for locoregional surgery in carefully selected patients.

## Data Availability

The data that support the findings of this study are available in the Surveillance, Epidemiology, and End Results (SEER) at https://seer.cancer.gov/. These data were derived from the following resources available in the public domain: Breast Cancer, https://seer.cancer.gov/data-software/documentation/seerstat/nov2024/.
